# Blood pressure and risk of cancer: a Mendelian randomization study

**DOI:** 10.1186/s12885-021-09067-x

**Published:** 2021-12-16

**Authors:** Io Ieong Chan, Man Ki Kwok, C. Mary Schooling

**Affiliations:** 1 School of Public Health, Li Ka Shing Faculty of Medicine, The University of Hong Kong, 7 Sassoon Road, Pokfulam, Hong Kong, SAR China; 2 Graduate School of Public Health and Health Policy, City University of New York, New York, USA

**Keywords:** Blood pressure, Cancer, Mendelian Randomization

## Abstract

**Background:**

Previous large observational cohort studies showed higher blood pressure (BP) positively associated with cancer. We used Mendelian randomization (MR) to obtain less confounded estimates of BP on total and site-specific cancers.

**Methods:**

We applied replicated genetic instruments for systolic and diastolic BP to summary genetic associations with total cancer (37387 cases, 367856 non-cases) from the UK Biobank, and 17 site-specific cancers (663–17881 cases) from a meta-analysis of the UK Biobank and the Kaiser Permanente Genetic Epidemiology Research on Adult Health and Aging. We used inverse-variance weighting with multiplicative random effects as the main analysis, and sensitivity analyses including the weighted median, MR-Egger and multivariable MR adjusted for body mass index and for smoking. For validation, we included breast (Breast Cancer Association Consortium: 133384 cases, 113789 non-cases), prostate (Prostate Cancer Association Group to Investigate Cancer Associated Alterations in the Genome Consortium: 79194 cases, 61112 non-cases) and lung (International Lung and Cancer Consortium: 10246 cases, 38295 non-cases) cancer from large consortia. We used asthma as a negative control outcome.

**Results:**

Systolic and diastolic BP were unrelated to total cancer (OR 0.98 per standard deviation higher [95% confidence interval (CI) 0.89, 1.07] and OR 1.00 [95% CI 0.92, 1.08]) and to site-specific cancers after accounting for multiple testing, with consistent findings from consortia. BP was nominally associated with melanoma and possibly kidney cancer, and as expected, not associated with asthma. Sensitivity analyses using other MR methods gave similar results.

**Conclusions:**

In contrast to previous observational evidence, BP does not appear to be a risk factor for cancer, although an effect on melanoma and kidney cancer cannot be excluded. Other targets for cancer prevention might be more relevant.

**Supplementary Information:**

The online version contains supplementary material available at 10.1186/s12885-021-09067-x.

## Background

Elevated blood pressure (BP), or hypertension, reduces population health globally [1], with 31.1% of the world’s adult population estimated to be hypertensive in 2010 [2], and 10.4 million deaths worldwide attributed to high systolic BP in 2016 [3]. In addition to the well-established relation of BP with cardiovascular disease (CVD) [4], hypertension has been linked with higher risk of cancer observationally [5–7], but the evidence is inconsistent with the possible exception of kidney cancer [8]. Secondary analyses of randomized controlled trials (RCTs) of antihypertensive drugs found little association with cancer [9], but RCTs typically have follow-up times too short to detect effects on cancer risk. Although the underlying mechanisms linking hypertension to cancer are still unclear, it has been suggested that increased cell turnover and telomere shortening could play a role [10]. In addition, dysregulated immune function is implicated in the pathogenesis of both hypertension and cancer [11, 12], and BP is positively associated with white blood cell count [13]. Nevertheless, confounding by social and environmental factors could give rise to the observed associations [14]. Mendelian randomization (MR), by using genetic variants randomly allocated at conception as instrumental variables, is less susceptible to confounding than conventional observational studies [15]. In this MR study using two-sample methods, we assessed the effects of systolic and diastolic BP on total cancer as well as on 17 common site-specific cancers, by applying replicated genetic instruments for BP to large population-based cohorts. For validation, we included large genetic consortia for breast, prostate and lung cancer. We also used multivariable MR [16, 17] to mitigate potential pleiotropic effects via obesity and smoking.

## Methods

### Genetic instruments for blood pressure

We extracted strong (*P* < 5x10^-8^), independent (*r*^2^ < 0.001) and externally replicated single nucleotide polymorphisms (SNPs) predicting BP from a meta-analysis of genome-wide association studies (GWAS) for BP traits totaling 757,601 participants of European ancestry (mean age 56.0 years, 54.7% women) [18], consisting of 458,577 individuals from the UK Biobank excluding pregnant women (*n*=372) and individuals who had withdrawn consent (*n*=36) [19], and 299,024 individuals from an enlarged dataset of the International Consortium for Blood Pressure (ICBP) with 77 cohorts [20]. Independent replication included 220,520 individuals from the Million Veteran Program [21] and 28,742 individuals from the Estonian Biobank of the Estonian Genome Center University of Tartu [22]. Participants on BP lowering medication had their BP values adjusted by adding 15 and 10 mm Hg to systolic and diastolic BP, respectively [23]. The UK Biobank analysis used a linear mixed model [24], adjusted for age, age^2^, sex and body mass index (BMI), with genomic control applied at the study level to correct for inflation due to population stratification and cryptic relatedness [25], followed by fixed-effect meta-analysis with the ICBP summary statistics which also adjusted for the same covariates. The pooled mean (standard deviation (SD)) systolic and diastolic BP were 138.4 (20.1) and 82.8 (11.2) mm Hg, respectively. The BP GWAS adjusted for BMI, which could bias the estimates of genetic variants on BP if genetic variants or environmental factors driving both BMI and BP exist [26], and potentially the MR estimates and/or instrument selection. We repeated the analysis for total cancer using genetic predictors from the UK Biobank, which did not adjust for BMI.

### Genetic associations with total and site-specific cancers

Genetic associations with total cancer (phenocode:195) were obtained from a pan-ancestry GWAS of the UK Biobank [27], with lifetime cancer occurrence ascertained from linked medical records (hospital inpatient data and death registry) including both prevalent and incident cases [28]. MR studies evaluate lifelong effects of an exposure, and so necessitate the inclusion of lifelong cases in consideration of potential selection bias [29]. Of the 441,331 participants included, genetic associations were provided for the 420,531 (95.3%) individuals of European ancestry to minimize confounding by population stratification. Non-cases were individuals without a diagnosis of primary or secondary cancer, nor a history of radio- or chemotherapy. The analyses used the Scalable and Accurate Implementation of Generalized mixed model, which accounts for sample relatedness and extreme case-control ratio [30], and adjusted for age, sex, age*sex, age^2^, age^2^*sex and the first 10 principal components (PCs).

Genetic associations with site-specific cancers were obtained from the largest available pan-cancer GWAS [31], which provides summary genetic associations with 17 cancers for 475,312 individuals of European ancestry from the UK Biobank and the Kaiser Permanente Genetic Epidemiology Research on Adult Health and Aging (GERA) [32, 33]. Lifetime cancer occurrence was ascertained from linked medical records with the latest diagnosis in August 2015 in the UK Biobank and June 2016 in GERA, which were converted into the third revision of International Classification of Diseases for Oncology (ICD-O-3) codes and classified according to organ site based on the U.S. National Cancer Institute Surveillance, Epidemiology, and End Results Program recode paradigm [34]. The median age at diagnosis was lowest for cervical cancer (37 and 38 years in the UK Biobank and GERA, respectively) and highest for pancreatic cancer (66 and 76 years). Individuals with multiple diagnoses were only recorded for their first cancer. Non-cases were cancer-free individuals, i.e., those who did not have any cancer diagnosis, self-reported history of cancer or cancer as a cause of death. For sex-specific cancer (breast, cervix, endometrium, ovary, prostate and testis), same-sex non-cases were used. Summary genetic associations are available for bladder, breast, cervix, colon, esophagus/stomach, kidney, lung, lymphocytic leukemia, melanoma, non-Hodgkin lymphoma, oral cavity/pharyngeal, ovary, pancreas, prostate, rectum and thyroid. The analyses were conducted separately for each cohort using logistic regression, adjusted for age, sex, the first 10 PCs, genotyping array (UK Biobank only) and reagent kit for genotyping (GERA only), followed by meta-analysis. Standard error (SE) of the SNP-outcome association were estimated from the *p*-value [35], as it was not provided.

For validation of potentially small effects, we additionally included large genetic consortia of leading cancers [36], i.e., breast (133384 cases and 113789 non-cases) [37], prostate (79194 cases and 61112 non-cases) [38] and lung (10246 cases and 38295 non-cases) [39], which have larger number of cases and do not overlap with the UK Biobank or GERA.

Estimates were aligned on the same effect allele for BP and cancer. Effect allele frequency (EAF) was not provided for pan-cancer, so we used the UK Biobank EAF which constituted 86% of the participants. Palindromic SNPs with ambiguous EAF, i.e. >0.42 and <0.58, and SNPs instrumenting BP but not available for an outcome, were replaced by proxies (*r*^2^ ≥ 0.8) identified using LDlink [40], wherever available.

### Genetic associations with asthma

Genetic associations with asthma (31169 cases and 379656 non-cases) used as a negative control outcome were obtained from the UK Biobank (phenocode: 495), given BP is not known to cause asthma [41], but both share similar confounders [42].

### Statistical analysis

Instrument strength was assessed using the F-statistic [43], approximated by the squared SNP-phenotype association divided by its variance. An F-statistic < 10 suggests potentially weak instrument. We also estimated the I^2^ to assess heterogeneity of instrument strength, an I^2^ < 90% suggests violation of the no measurement error (NOME) assumption and possibly invalid estimates [43]. An I^2^ > 97% suggests minimal bias of the MR estimates by confounding of exposure on outcome in overlapping samples [44], as here. The proportion of phenotypic variance (r^2^) explained by the genetic instruments was calculated as beta^2^*2*MAF*(1-MAF), where beta is the SNP-phenotype association standardized to the phenotypic variance and MAF is the minor allele frequency of the SNP [45]. Power calculations were based on the approximation that the sample size for an MR study is the sample size for exposure on outcome divided by the r^2^ for genetic instruments on exposure [46], using an online tool [47].

We used the inverse-variance weighted (IVW) meta-analysis, with multiplicative random effects, which assumes balanced pleiotropy [48], of the SNP-specific Wald estimates, i.e., the SNP-outcome association divided by the SNP-exposure association, as the main analysis. We also conducted sensitivity analyses using the weighted median [49] and MR-Egger [50]. The weighted median assumes 50% of the weight is from valid SNPs. MR-Egger is robust to genetically invalid instruments given the instrument strength Independent of direct effect (InSIDE), i.e., the instruments do not confound exposure on outcome, and the NOME assumption is satisfied. A zero MR-Egger intercept indicates evidence of lack of such genetic pleiotropy. Some of the genetic instruments for BP was previously shown to be associated with confounders of BP and cancer, mostly for anthropometrics and a few for lifestyle [18], so we used multivariable MR to estimate the effects of BP on cancer independent of BMI or ever-smoking using IVW or MR-Egger if the intercept was non-zero. We obtained genetic associations with BMI and ever-smoking from Yengo et al. [51], and the Social Science Genetic Association Consortium [52], respectively. We dropped correlated predictors of BP and BMI or ever-smoking. We estimated the Sanderson-Windmeijer multivariate F-statistic and modified Q statistic to assess conditional instrument strength and heterogeneity, taking into account the phenotypic correlation [53], using estimates from the UK Biobank [54].

All analyses were performed using R (version 4.0.1, The R Foundation for Statistical Computing Platform, Vienna, Austria). We used the R packages “TwoSampleMR”, “MedelianRandomization” and “MVMR”. Given the number of cancer outcomes considered, a two-sided p-value below the Bonferroni-corrected significance threshold 0.0014 (0.05/2 BP traits*18 cancer outcomes) was used.

## Results

There were 272 and 267 strong, independent and replicated SNPs predicting systolic (Supplementary Table S1) and diastolic (Supplementary Table S2) BP with a mean (range) F-statistic of 83.2 (29.3 – 612.4) and 90.7 (30.0 – 818.1), and I^2^ of 92.5 and 93.8%, respectively (Table 1). These SNPs explained approximately 2.59 and 2.96% of the variance of systolic and diastolic BP, respectively. At 5% alpha, this study has 80% power to detect an odds ratio (OR) of about 1.09 for total cancer, and from 1.13 for breast to 1.60 for thyroid cancer per SD of BP (Supplementary Table S3). We obtained 539 and 92 strong (*P* < 5x10^-8^) and independent (*r*^2^ < 0.001) SNPs predicting BMI and ever-smoking, respectively. Both BMI (Supplementary Fig. S1) and ever-smoking (Supplementary Fig. S2) were positively associated with total cancer. The Sanderson-Windmeijer multivariate F-statistics were at least 33.8 for systolic and 36.9 for diastolic BP when adjusted for BMI, and at least 75.3 for systolic and 80.8 for diastolic BP when adjusted for ever-smoking.

**Table 1:** Genome-wide association studies of total and 17 site-specific cancers

Outcome	No. of SNPs used (systolic/diastolic)	Total sample size	No. of cases			No. of non-cases		
			UK Biobank	GERA	Total	UK Biobank	GERA	Total
Total cancer	272/267	405243	37387	-	-	367856	-	-
Bladder	270/267	412592	1550	692	2242	359825	50525	410350
Breast	269/266	237537	13903	3978	17881	189855	29801	219656
Cervix	270/267	226219	5998	565	6563	189855	29801	219656
Colon	270/267	414143	2897	896	3793	359825	50525	410350
Endometrium	270/267	221693	1414	623	2037	189855	29801	219656
Esophagus/stomach	270/267	411441	929	162	1091	359825	50525	410350
Kidney	270/267	411688	1021	317	1338	359825	50525	410350
Lymphocytic Leukemia	270/267	411202	594	258	852	359825	50525	410350
Lung	270/267	412835	1728	757	2485	359825	50525	410350
Melanoma	270/267	417127	4271	2506	6777	359825	50525	410350
Non-Hodgkin's Lymphoma	270/267	412750	1760	640	2400	359825	50525	410350
Oral cavity/pharyngeal	270/267	411573	930	293	1223	359825	50525	410350
Ovary	270/267	220915	1006	253	1259	189855	29801	219656
Pancreas	270/266	411013	471	192	663	359825	50525	410350
Prostate	268/267	201486	7441	3351	10792	169970	20724	190694
Rectum	270/267	412441	1808	283	2091	359825	50525	410350
Thyroid	270/267	411112	527	235	762	359825	50525	410350

*Abbreviations*: *GERA* Kaiser Permanente Genetic Epidemiology Research on Adult Health and Aging, *SNP* single nucleotide polymorphism

**Fig. 1:**
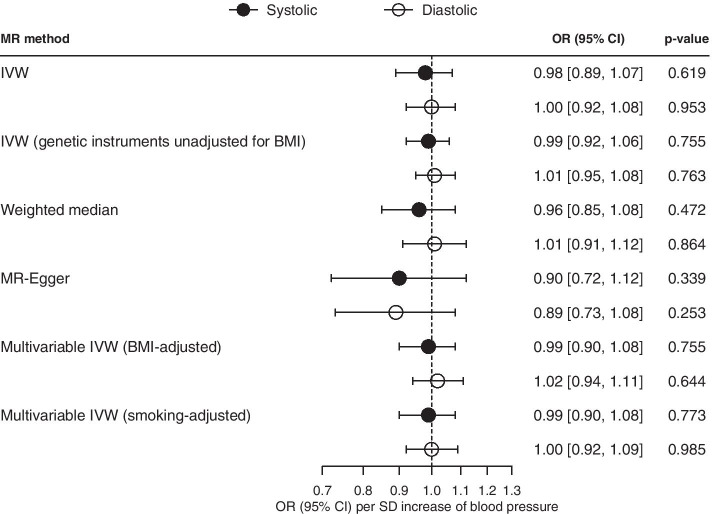
Mendelian randomization (MR) estimates of systolic and diastolic BP on total cancer. BMI, body mass index; IVW, inverse variance weighting

Figure 1 shows the associations of systolic and diastolic BP (per 1-SD increment) with total cancer. Overall, systolic (OR 0.98 [95% confidence interval (CI) 0.89, 1.07] and diastolic BP (OR 1.00 [95% CI 0.92, 1.08]) were not associated with total cancer. Repeating the analysis using genetic instruments unadjusted for BMI, or sensitivity analysis using the weighted median, MR-Egger and multivariable MR gave similar results (Supplementary Tables S4 and S5).

**Fig. 2:**
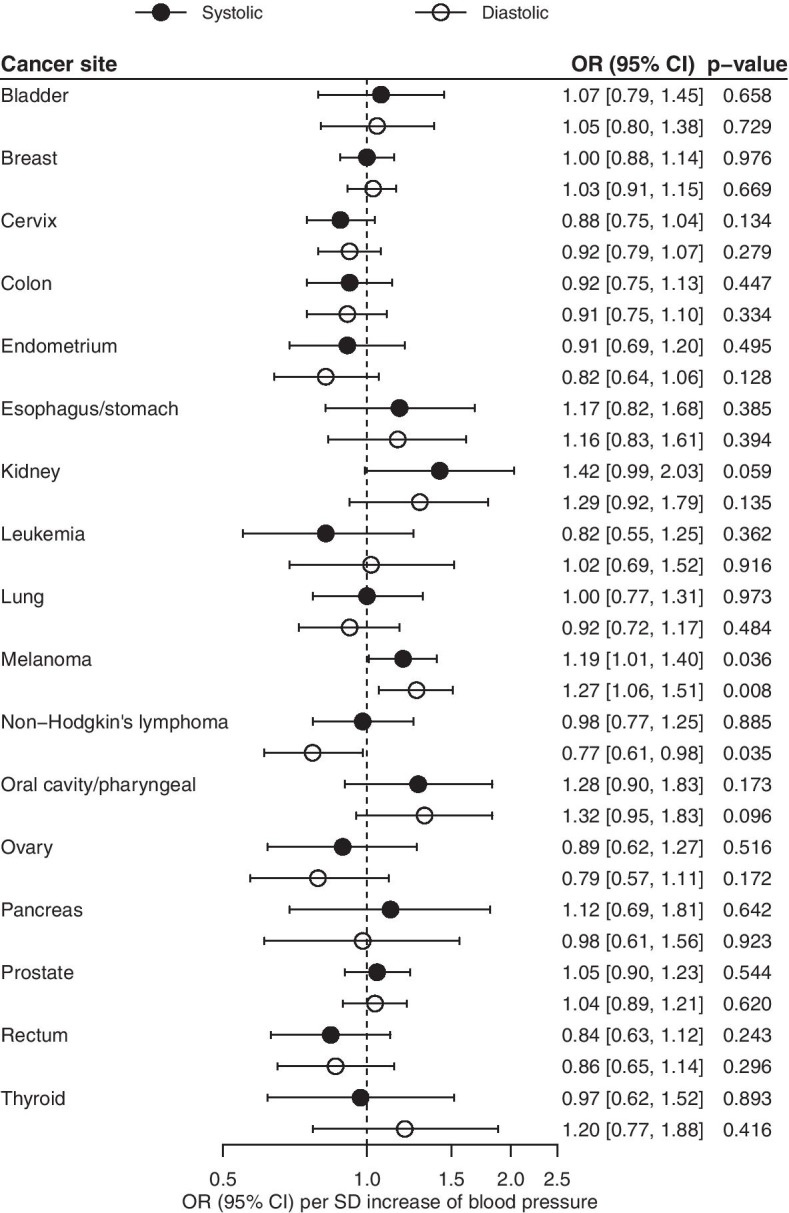
Inverse-variance weighted Mendelian randomization estimates of systolic and diastolic BP on 17 site-specific cancer

**Fig. 3:**
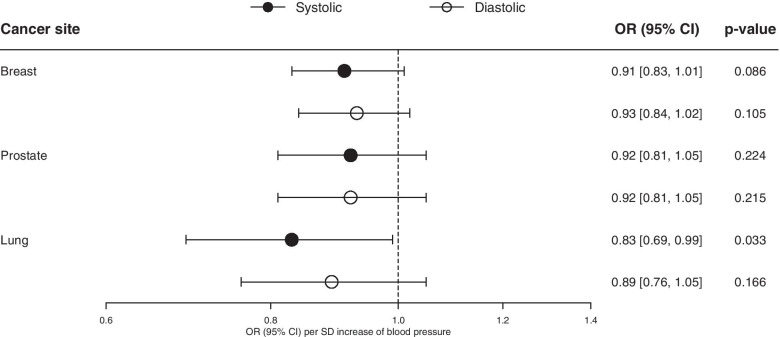
Inverse-variance weighted Mendelian randomization estimates of systolic and diastolic BP on breast, prostate and lung cancers in genetic consortia

Systolic and diastolic BP were not significantly associated with any of the 17 site-specific cancers in the meta-analysis of the UK Biobank and GERA. Some associations at nominal significance were observed for kidney cancer and melanoma (Fig. 2). Similarly, no significant associations were observed for breast, prostate or lung cancer (Fig. 3) in the consortia, although systolic BP was nominally associated with lung cancer. Using other MR methods gave similar results for site-specific cancers (Supplementary Tables S4-6). Systolic and diastolic BP were not associated with asthma (Supplementary Fig. S3).

## Discussion

Consistent with secondary analyses of RCTs [9], but less consistent with observational studies [5–8], this MR study found little evidence of BP increasing risk of cancer. However, BP was nominally positively associated with kidney cancer [55, 56], and possibly melanoma [57]. As expected, BP was not associated with asthma.

This is the first MR study that has comprehensively evaluated the effect of BP on cancer. The main strength of the study is the MR design which minimizes confounding [58]. Long-term exposure to common risk factors for cancer [14], such as socio-economic position and all it entails, including smoking [59], alcohol consumption [60], diets promoting obesity [61], and air pollution [62] are known to elevate BP. So, previous observational findings showing higher BP positively associated with risk of total and some site-specific cancers [5–8], might be due to confounding by these factors. Sustained hypertension leads to compensatory vascular hypertrophy involving Angiotensin II mediated by various growth factors [63]. Angiotensin II receptors are found in high density in the kidney responsible for BP regulation [64]. A previous MR showed diastolic BP, but not systolic BP, was associated with higher risk of kidney cancer [56]. We did not replicate these findings at statistical significance, but only with directionally concordant results. Here, we used a different GWAS for kidney cancer, which had fewer cases, and adjusted for more covariates, such as age, to control for population structure. Further studies with more cases of kidney cancer would be helpful. Similarly, tumour growth following melanoma cell grafting was slower in Angiotensin II receptor deficient mice than in wild types [65]. Observationally, BP was positively associated melanoma [57], and further MR studies with larger samples are warranted. Although MR is less susceptible to confounding than traditional observation studies, it is not free from selection bias [66]. Specifically, given BP strongly reduces survival, some MR estimate may be attenuated by missing people who died before recruitment from genetically higher BP, from cancer or from a competing risk for cancer [67], such as CVD, particularly for cancers typically identified at older ages, including kidney cancer, prostate cancer and melanoma. As such, the estimate for total cancer could be a false negative. However, most cancer deaths typically occur at a younger age than deaths from other major causes [68], such as CVD, reducing this possibility.

Despite using a design less open to confounding than purely observational studies, and assessing associations independent of BMI as well as of smoking, our study has several limitations. First, the validity of MR rests on the three instrumental variable assumptions, i.e., the genetic instruments strongly predict the exposure, the genetic instruments are not associated with confounders of the exposure and outcome, and the genetic instruments are associated with the outcome only through affecting the exposure [69]. We used the largest available GWAS with external replication to obtain genetic instrument for BP, and sensitivity analysis to assess the robustness of our estimates, which were largely consistent. We also included a negative control outcome and did not find evidence of substantial pleiotropic effects. Second, the UK Biobank contributed information to the exposure and outcome GWAS, which may bias the MR estimates towards the observational association [70] particularly for weak instruments. However, weak instrument bias is inversely proportional to the F-statistics, which was only around 1%. Bias from confounding is unlikely to affect the analysis and would not explain the null findings [44]. Third, total cancer was based on incident and prevalent cases which might over-represent people living with treatable cancers. However, cancers have common underlying molecular hallmarks [71], whether BP might affect these hallmarks is unclear. In addition to total cancer, we investigated the effects of BP on 17 site-specific cancers. Although we found no association of BP with total cancer, we cannot rule out the possibility that BP has some specific effects on some site-specific cancers, which we could not reliably test owing to the small number of cases for some cancers. Furthermore, the grouping of heterogenous cancer sites, such as esophagus and stomach, limited the interpretation of some of our estimates, but were included for completeness. We additionally included large genetic consortia for breast, prostate and lung cancer for validation. Notably, these GWAS did not adjust for age, and the cases were on average younger than the non-cases, which could confound the MR estimates likely away from the null. Fourth, the BP instruments were adjusted for BMI. Adjusting for an effect of the exposure does not necessarily create bias [72], correspondingly genetic estimates for BP have been shown to be similar with and without adjustment for BMI [73]. Using genetic instruments for BP without adjustment for BMI also made little difference to the estimate for total cancer. Fifth, the UK Biobank is self-selected and differs from its underlying population in several major health and socioeconomic characteristics [74]. However, risk factor-outcome associations are comparable in the UK biobank and other UK-based studies with less self-selection [75]. Sixth, the present study included only participants of European ancestry, which avoids genetic confounding due to population stratification but may limit external validity in other ethnic groups. However, BP is not thought to act differently by ethnicity [76].

Globally, BP has been falling, most notably in high sociodemographic index countries in Asia Pacific and the West [77]. However, cancer rates are still rising even after taking into account population aging [78]. Obesity prevalence has been rising in both children and adults [79], which may instead underlie some of the rising cancer incidence, as well as raising BP. From a population health perspective, our findings are largely consistent with the absence of hypertension as an intervention target for primary cancer prevention [80]. Although this may undermine the importance of hypertension as a risk factor for health, it is perhaps more important that the benefits of BP lowering be accurately mapped out for evidence-based health promotion [81].

## Conclusions

In this MR study, BP does not appear to be a risk factor for total cancer contrary to previous observational evidence, although an effect on melanoma and kidney cancer cannot be excluded. Other targets for cancer prevention might be more relevant.

## Supplementary Information

**Additional file 1: Supplementary Figure S1**. Mendelian randomization estimates of body mass index on total cancer.

**Additional file 2: Supplementary Figure S2.** Mendelian randomization estimates of ever-smoking on total cancer.

**Additional file 3: Supplementary Figure S3.** Mendelian randomization estimates of systolic (filled) and diastolic (hollow) blood pressure on asthma.

**Additional file 4: Supplementary Table S1**. Genetic associations with systolic blood pressure, total and site-specific cancers. **Supplementary Table S2**. Genetic associations with diastolic blood pressure, total and site-specific cancers. **Supplementary Table S3**. Power calculations for total and site-specific cancers. **Supplementary Table S4**. Mendelian randomization estimates of systolic (per 10 mmHg increment) blood pressure on total and site-specific cancers. **Supplementary Table S5**. Mendelian randomization estimates of diastolic (per 5 mmHg increment) blood pressure on total and site-specific cancers. **Supplementary Table S6**. Mendelian randomization estimates of systolic (per 10 mmHg increment) and diastolic (per 5 mmHg increment) blood pressure on site-specific cancers in genetic consortia

## Data Availability

We thank the participants and researchers for providing the publicly available summary data used in this study. Data on blood pressure were downloaded from GWAS Catalog (ebi.ac.uk/gwas/); data on total cancer were downloaded from the UK Biobank (pan.ukbb.broadinstitute.org/); data on site-specific cancers were downloaded from github.com/Wittelab/pancancer_pleiotropy; data on breast cancer were downloaded from bcac.ccge.medschl.cam.ac.uk; data on prostate cancer were downloaded from practical.icr.ac.uk; data on lung cancer were downloaded from gwas.mrcieu.ac.uk; data on BMI were downloaded from portals.broadinstitute.org/collaboration/giant; data on ever-smoking were downloaded from the Social Science Genetic Association Consortium (thessgac.org).

## Abbreviations

BMI: Body mass index
BP: Blood pressure
CI: Confidence interval
CVD: Cardiovascular disease
EAF: Effect allele frequency
GERA: Kaiser Permanente Genetic Epidemiology Research on Adult Health and Aging
GWAS: Genome-wide association studies
ICBP: International Consortium for Blood Pressure
ICD-O-3: Third revision of International Classification of Diseases for Oncology
IVW: Inverse-variance weighted
InSIDE: Instrument strength Independent of direct effect
MAF: Minor allele frequency
MR: Mendelian randomization
NOME: No measurement error
OR: Odds ratio
PC: Principal component
RCT: Randomized controlled trial
SD: Standard deviation
SE: Standard error
SNP: Single nucleotide polymorphism
