# Supplementary material for: Blood pressure and risk of cancer: a Mendelian randomization study
Source: BMC Cancer. 2021 Dec 16;21:1338. doi: 10.1186/s12885-021-09067-x (PMC8675492; doi:10.1186/s12885-021-09067-x)
Supplement: Supplementary file 2 — Additional file 2: Supplementary Figure S2. Mendelian randomization estimates of ever-smoking on total cancer. [file 12885_2021_9067_MOESM2_ESM.docx]

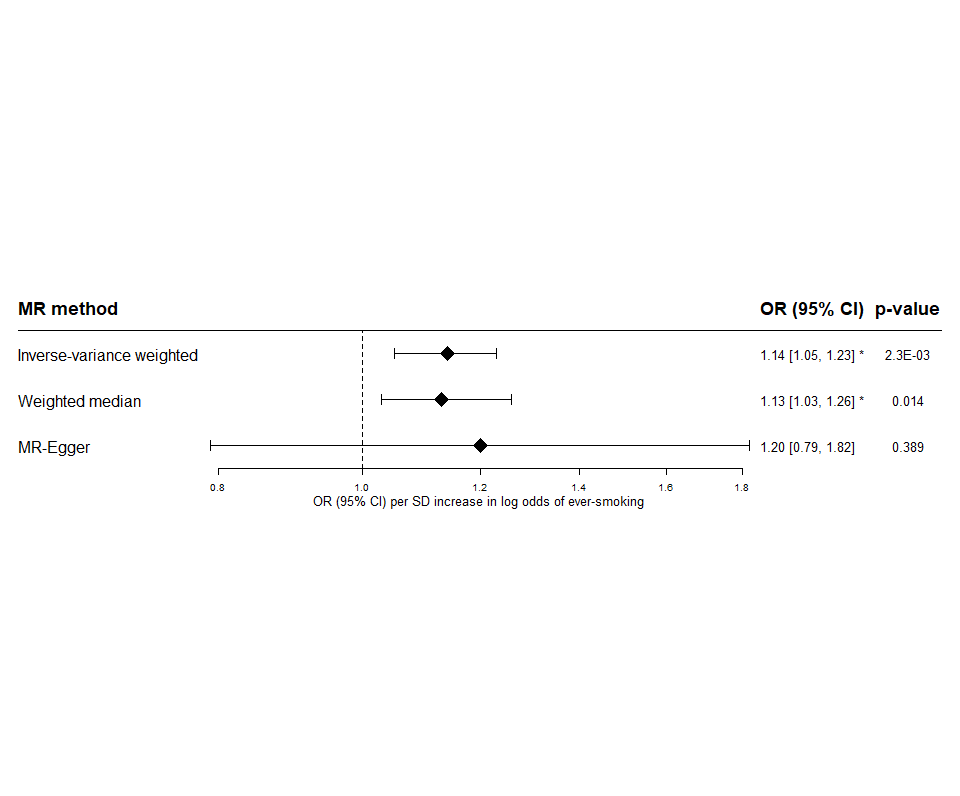


**Supplementary Figure S2. Mendelian randomization estimates of ever-smoking on total cancer. P for MR-Egger intercept = 0.791.**
