# Supplementary material for: Blood pressure and risk of cancer: a Mendelian randomization study
Source: BMC Cancer. 2021 Dec 16;21:1338. doi: 10.1186/s12885-021-09067-x (PMC8675492; doi:10.1186/s12885-021-09067-x)
Supplement: Supplementary file 3 — Additional file 3: Supplementary Figure S3. Mendelian randomization estimates of systolic (filled) and diastolic (hollow) blood pressure on asthma. [file 12885_2021_9067_MOESM3_ESM.docx]

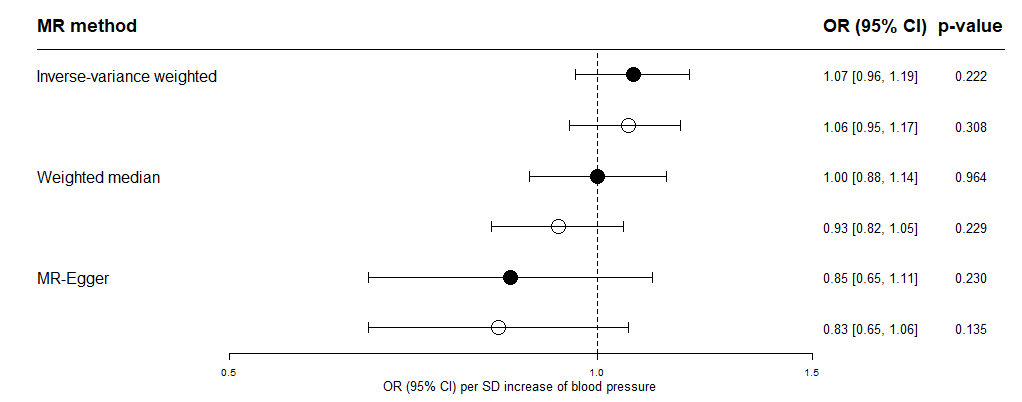


**Supplementary Figure S3. Mendelian randomization estimates of systolic (filled) and diastolic (hollow) blood pressure on asthma.**
